# Nondestructive Prediction of Isoflavones and Oligosaccharides in Intact Soybean Seed Using Fourier Transform Near-Infrared (FT-NIR) and Fourier Transform Infrared (FT-IR) Spectroscopic Techniques

**DOI:** 10.3390/foods11020232

**Published:** 2022-01-16

**Authors:** Hanim Z. Amanah, Salma Sultana Tunny, Rudiati Evi Masithoh, Myoung-Gun Choung, Kyung-Hwan Kim, Moon S. Kim, Insuck Baek, Wang-Hee Lee, Byoung-Kwan Cho

**Affiliations:** 1 Department of Biosystems Machinery Engineering, College of Agricultural and Life Science, Chungnam National University, 99 Daehak-ro, Yuseong-gu, Daejeon 34134, Korea; Hanim_za@ugm.ac.id (H.Z.A.); sstunny@o.cnu.ac.kr (S.S.T.); wanghee@cnu.ac.kr (W.-H.L.); 2 Department of Agricultural and Biosystems Engineering, Faculty of Agricultural Technology, Gadjah Mada University, Yogyakarta 55281, Indonesia; evi@ugm.ac.id; 3 Department of Herbal Medicine Resource, Dogye Campus, Kangwon National University, Samcheok 25949, Korea; cmg7004@kangwon.ac.kr; 4 Department of Gene Engineering, National Institute of Agricultural Sciences, Rural Development Administration, Jeonju 54874, Korea; biopiakim@korea.kr; 5 Environmental Microbial and Food Safety Laboratory, Agricultural Research Service, United States Department of Agriculture, Powder Mill Road, BARC-East, Bldg 303, Beltsville, MD 20705, USA; moon.kim@usda.gov (M.S.K.); insuck.baek@usda.gov (I.B.); 6 Department of Smart Agriculture Systems, College of Agricultural and Life Science, Chungnam National University, 99 Daehak-ro, Yuseong-gu, Daejeon 34134, Korea

**Keywords:** isoflavones, oligosaccharides, soybean seed, spectroscopic techniques

## Abstract

The demand for rapid and nondestructive methods to determine chemical components in food and agricultural products is proliferating due to being beneficial for screening food quality. This research investigates the feasibility of Fourier transform near-infrared (FT-NIR) and Fourier transform infrared spectroscopy (FT-IR) to predict total as well as an individual type of isoflavones and oligosaccharides using intact soybean samples. A partial least square regression method was performed to develop models based on the spectral data of 310 soybean samples, which were synchronized to the reference values evaluated using a conventional assay. Furthermore, the obtained models were tested using soybean varieties not initially involved in the model construction. As a result, the best prediction models of FT-NIR were allowed to predict total isoflavones and oligosaccharides using intact seeds with acceptable performance (*R*^2^*_p_*: 0.80 and 0.72), which were slightly better than the model obtained based on FT-IR data (*R*^2^*_p_*: 0.73 and 0.70). The results also demonstrate the possibility of using FT-NIR to predict individual types of evaluated components, denoted by acceptable performance values of prediction model (*R*^2^*_p_*) of over 0.70. In addition, the result of the testing model proved the model’s performance by obtaining a similar *R*^2^** and error to the calibration model.

## 1. Introduction

The global market related to functional food has been increasing considerably in the last decade. In Asia, the market is predicted to continue to grow due to the high population [1]. In terms of functional foods, soybeans are one of the most interesting products to be evaluated due to the complex chemical compound inside. Soybeans are one of the world’s most protein sources and are the primary protein supply in animal feed. It is also reported as a leading share in the global oilseed output and contributes to 60% of the world’s meal production. Wilson in Roger-Boerma [2] reported that protein and oil content in soybean ranges from 341 to 568 g/kg (34.1–56.8%) and 83 to 279 g/kg (8.3–27.9%) of total seed weight, respectively. The remaining macronutrient in soybean is carbohydrates (including dietary fiber and soluble carbohydrates), which account for about 35% of a moisture-free soybean [3].

The composition of soybean carbohydrates was already described in detail by Lilienthal et al. [4] On average, mature soybeans contain 15% oligosaccharides, and about 50% of them (6–7%) are sucrose. The raffinose families (stachyose, raffinose, and verbascose) are available in 4–5%, 1–2%, and below 1%, respectively. It has been a long time since soybean oligosaccharides were considered to cause flatulence [5], but they have also been approved as a prebiotic material to improve the quality of microflora in the human intestine [6]. Due to their economic value to the food and feed industries, efforts to recover these components from defatted soybean meal have been carried out. For example, the sweetener industry has applied new technology to make a commercialized high-oligosaccharides syrup from soybean. Moreover, the attractive effect of galactooligosaccharides in soybean has also arrested the attention of researchers to perform an in-depth evaluation for future commercialization.

Despite containing interested oligosaccharides components, soybean also contains isoflavones that benefit human health. The chemical structure of isoflavones, which have a phenolic structure similar to estrogen, allows them to bind to estrogen receptors and exhibit estrogenic activity [7,8]. Moreover, isoflavones and dietary food containing high isoflavones concentrations have been reported as possible anticarcinogens. In addition, several works and reviews reported the relationship between isoflavones and the relief of chronic diseases in humans such as prostate cancer [9], obesity and diabetes [10,11], osteoporosis [12], and the incidence of cardiovascular disease [13]. These studies have opened doors to increase the consumption of traditional soy-based food (soy milk, tofu, natto, tempeh, etc.) and modern food with soy-based ingredients. Furthermore, commercial isolate soy isoflavones are available in the market as pills, concentrates, and extracts.

The increased demand for bioactive compounds encourages the breeding industry to produce enriched new lines of seeds to fulfill consumer demands. Therefore, seed selection will play a key role in providing high-quality seed related to the targeted bioactive components. On the other hand, controlling the quality of enriched bioactive compound products before reaching the consumer’s hand is also crucial to avoid excessive producer claims. From this point of view, a precise and appropriate technique to evaluate the content of the specified biological components is necessary.

Modern laboratories in chemistry have approached many precise measurement methods to determine the chemical composition inside the sample, including high-performance liquid chromatography (HPLC), mass spectrometry (MS), and capillary electrophoresis (CE). These methods have proved their preciseness and reproducibility in determining the macro- and micro-chemical components of soybeans [14,15,16,17,18,19]. However, those methods cannot address environmental issues related to the high amount of chemical waste, as well as being highly cost- and labor-intensive. In addition, these methods destroy the sample during the preparation process prior to analysis, which meets the limitation of the breeding company in the seed-selection process. On the other hand, the determination of seed quality is a meaningful consideration for producers to achieve successful production [20]. Hence, the development of nondestructive techniques is demanding.

One such nondestructive approach combines vibrational spectroscopic techniques with chemometric analysis, successfully applied in food and agricultural products’ qualitative and quantitative determination. For example, MIR spectroscopy has been used to predict sugar in apple juice [21] and detect hairtail surimi in mixed surimi [22]. On the other hand, NIR has also been successfully used to determine the quality parameter of maize including protein, carbohydrate, oil, and ash [23]. Furthermore, Masithoh et al. [24] compare the effectiveness of NIR and MIR to determine protein and glucose content in the tuber and root flour. In the case of soybean, NIR and MIR have been used to determine soybean important components, including protein, moisture, lipids, and ash [25,26], and micro components such as saponins, fatty acids, flavonoids, and isoflavonoids [27]. In addition, the two most common anthocyanins in soybeans have also been successfully predicted based on the spectra of NIR [28]. However, all of the previous research for soybean collected spectral data using a powder sample type.

An investigation of soybean chemical components based on the data obtained from seed samples has also been carried out. Kovalenko et al. [29] evaluated amino acids content in soybean based on NIR spectral data of the seeds. Moreover, three different sample types (single-bean, whole bean, and powder) have been investigated to determine their effects on the success of predicting protein and amino acids in soybeans [30], while FT-NIR and FT-IR (in MIR region) spectra from intact seed have also been successfully used to predict anthocyanin in soybean [31]. The reports reveal that using a single bean to obtain NIR and MIR spectral data is a promising strategy for nondestructive chemical component evaluation of soybeans. However, soybean protein and amino acid content are relatively high (about 40% dw), and anthocyanin is only found on the bean surface.

Our concern in this study was soybean isoflavones and oligosaccharides, which are available in low concentrations and distributed in the whole bean part. Therefore, the effectiveness of FT-NIR and FT-IR was investigated to predict the above-mentioned targeted components based on the spectral data of a single bean. In addition, a more detailed investigation of the individual type of each microcomponent has also been carried out and presented side-by-side with the results of powder samples.

## 2. Materials and Methods

### 2.1. Sample Preparation

In this research, 310 soybean samples were used to develop models to predict the examined soybean components. These samples were supplied by Rural Development Administration (RDA), South Korea, and among them, seventy samples had black color coats. The bean size was varied based on the sample ranging from 2.90 to 7.90 mm. In addition, the bean size within a single sample was also varied, which possibly affected the chemical content among different seeds [32]. Therefore, the seed’s chemical composition variability will affect the spectral data measured by every instrument. On the other hand, the evaluation of chemical components was constrained by the minimum weight requirement in the extraction process prior to the HPLC process. Hence, in this study, 30 g of soybean seeds were collected from each examined sample, and among them, twenty-one beans were selected randomly for the spectral data acquisition using Fourier transform near-infrared (FT-NIR) and Fourier transform infrared (FT-IR) systems. Therefore, there were 6510 (21 bean × 310 samples) spectral data collected for every instrument, which were then divided into calibration and validation datasets about 70% and 30%, respectively. After being measured using both instruments, all 30 g of beans were ground, obtaining powder that passed through the sieve designation mesh no 60, with a sieve opening at 250 microns. The spectral data for the powder samples were then collected using both instruments in the same instrumental setup. Finally, the powder samples were sent for chemical analysis to obtain reference values for further analysis.

The additional 65 soybean samples containing yellow soybeans (50 samples) and black soybean (15 samples), which were not initially involved in the model construction and obtained from the same institution, were used for testing the resulting model. Twenty-one beans were also selected randomly from each sample and collected their spectral data using both instruments.

### 2.2. Spectral Data Acquisition

#### 2.2.1. FT-NIR Spectroscopy

**Figure 1:**
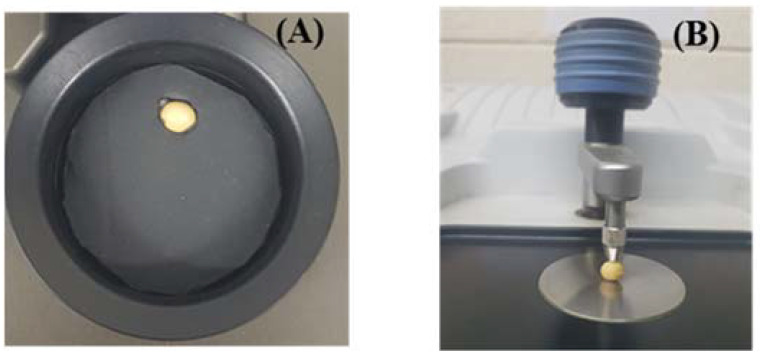
The photograph of the bean seed spectral data acquisition of FT-NIR (**A**) and FT-IR (**B**).

Laboratory FT-NIR spectrometer Antaris II FT-NIR analyzer (Thermo Scientific Co. Waltham, MA, USA) was performed to acquire the diffuse reflectance spectral data of soybean seed in range of waveband between 10,000 and 4000 cm^−1^ (1000–2500 nm). A single bean was placed in the sample holder and scanned 32 times to obtain average spectral data in the specified waveband at 4 cm^−1^ intervals. The interference of environmental light during the measurement process was blocked by covering the sample holder with a black lid (Figure 1A). Similar techniques and instrumental settings were applied to collect spectral data of powder samples.

#### 2.2.2. FT-IR Spectroscopy

The spectra of soybean seeds and powder samples were collected using an FT-IR spectrometer Nicolet 6700 (Thermo Scientific Co., Waltham, MA, USA) equipped with an attenuated total reflectance (ATR) sampling mode. The absorbance spectra of each seed were collected in the MIR wavelength region ranging from 4000 to 400 cm^−1^ (2500–25,000 nm) at the interval of 4 cm^−1^ spectral resolution. During the measurement, soybean seed was placed on the surface of the diamond crystal and clamped using a pointed tip (Figure 1B), while the background was collected by scanning an empty plate. As a result, the average spectrum of 32 successive scans of each seed was obtained for further analysis. In this study, the FT-IR with an ATR sampling model was selected due to only requiring a very small quantity of sample [33], allowing the analysis of the sample in its natural state. Moreover, the FT-IR ATR also enables us to measure directly onto a solid-state sample surface by pressing the sample towards an ATR crystal with no sample preparation.

### 2.3. Reference Value Investigation

#### 2.3.1. Chemicals

Pure water (18 MΩ cm^−1^) was obtained from a Milli-Q water purification system (Millipore, Bedford, MA, USA). HPLC analytical grade water, methanol, ethanol, acetonitrile, and acetic acid were obtained from J.T. Baker Co (Phillipsburg, NJ, USA). Oligosaccharide standards, as stachyose trihydrate, raffinose, and sucrose, were obtained from Sigma Chemical Co. (St. Louis, MO, USA). All isoflavone standards, 6″-O-malonyl daidzin, 6″-O-malonyl glycitin, 6″-O-malonyl genistin, 6″-O-acetyl daidzin, 6″-O-acetyl glycitin, 6″-O-acetyl genistin, daidzin, glycitin, genistin, daidzein, glycitein, and genistein were purchased from Carbosynth company (Compton, Berkshire, UK). All other laboratory chemicals used in this study were reagent grade.

#### 2.3.2. Isoflavones Determination

The isoflavones determination was carried out based on the procedure used by previous researchers [34] with some modifications. One gram of soybean sample was extracted with 70% EtOH (40 mL) at room temperature for 24 h. The 70% EtOH extract was filtered with Whatman No. 6 (Whatman Inc., Maidstone, UK) filter paper. The 20 mL of 70% EtOH extract was evaporated under 40 °C and dissolved in 70% EtOH (5 mL). Before analysis, all samples were filtered through a 0.45 µm membrane filter (Whatman Inc., Maidstone, UK)

An Agilent 1260 series HPLC system (Wilmington, DE, USA) equipped with a photodiode array detector operating at 260 nm was used for analysis. Separations were performed on a Venusil C18 column (5 µm; 250 × 4.6 mm i.d., Agela Technologies, Torrance, CA, USA) protected with a Nova-Pak C18 guard insert column (Waters, Milford, MA, USA). The column temperature was maintained at 30 °C with the mobile phases consisting of solvent (A): 0.1% (*v*/*v*) acetic acid in the water, and solvent (B): 0.1% (*v*/*v*) acetic acid in acetonitrile.

The gradient elution profile was as follows: 0 min, 85% A, 0–60 min, 85–70% A; 60–65 min, 70–60% A; 66 min, 85% A; 66–75 min, 85% A. The flow rate was 1.0 mL/min, and the injection volume was 20 µL. The isoflavone contents were calculated by comparing HPLC peak areas with external standard calibration curves. The linear standard calibration curves (*R*^2^ = 0.999) were generated by injecting 0–20 µg of isoflavone standards in 1 mL of 80% methanol.

#### 2.3.3. Oligosaccharides Determination

In brief, soybean powder (1.0 g) was extracted with 70% EtOH (10 mL) at room temperature for 24 h and then centrifuged at 5000× *g* for 20 min. Next, the supernatant was filtered with a Sep-Pak C18 solid-phase extraction cartridge (Waters, Milford, MA, USA), and the remaining residue was then dissolved in water. Finally, the diluted extract (20 µL) was injected into an HPLC system.

Oligosaccharides were analyzed using an Agilent 1200 series (Wilmington, DE, USA) HPLC system with Sedex 75 ELSD (Evaporative Light Scattering Detector, Sedere, Alfortville, France). The diluted extract (20 µL) was injected into an HPLC system equipped with a Shodex HILICpak VG-50 4E (4.6 × 250 mm, 5 μm, Showa Denko Co., Tokyo, Japan) column. The column temperature was 30 °C, and the mobile phase (flow rate 1.5 mL/min) was a linear gradient prepared from acetonitrile (A) and water (B). The gradient program was (time,% A): 0–20 min, 80–50%; 21–27 min, 20%; 28 min, 80%; 28–38 min, 80%. The temperature for the flow shift tub in ELSD was 45 °C. The flow rate of N2 was 1.0 bar. The contents of oligosaccharides were calculated by comparing the HPLC peak areas with the external standard calibration curves. The standard calibration curves (*R*^2^ = 0.999) were generated by injecting 0–300 mg of three oligosaccharide standards in 1 mL of water.

### 2.4. Spectral Data Preprocessing and Multivariate Analysis

All spectral data collected using FT-NIR and FT-IR were subjected to several preprocessing methods, including normalization (mean, range, and max), standard normal variate (SNV); Multiplicative Scatter Correction (MSC); and Savitzky-Golay (SG) first and second derivative methods. This process aims to remove noise generated during the measurement due to instrumental or environmental effects [35,36]. The obtained preprocessed spectral data were then divided into calibration and validation datasets by 70% and 30%, respectively.

Afterward, a multivariate analytical model of partial least square regression (PLS-R) was selected to develop models to predict the content of the targeted components. The general equation for PLS-R is expressed as follows:

(1) Y=X×B+E

where *X* is an m×n matrix that holds the spectra values of the sample, B is the regression coefficient, and E is the error term. In this study, for the construction of the PLS-R model, spectral data of soybean seeds and powder were arranged in a matrix *X*, while the *Y* matrix contained the reference values obtained from chemical analysis. The values *X* and *Y* are decomposed into latent variables (LVs) to establish a linear connection between the response and predictor variables. The optimum number of LVs was selected based on the standard procedure documented by Varmuza and Filzmozer [37] Section 4.2.

(2) $$X=TP^{T}+E_{x}$$

(3) $$Y=UQ^{T}+E_{y}$$

**Figure 2:**
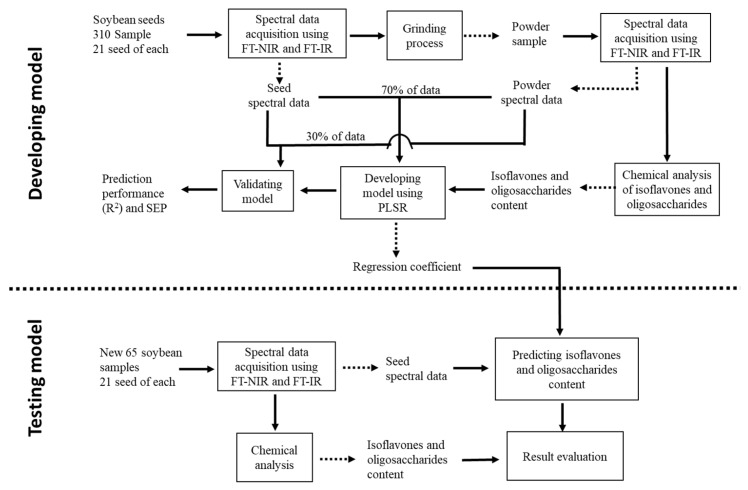
The experimental workflow to develop a model to predict isoflavones and oligosaccharides using FT-NIR and FT-IR spectroscopic techniques.

*T* and *U* are score matrices, whereas *P* and *Q* are loading matrices. E_x_ and E_y_ are the error matrix of *X* and *Y*, respectively. Statistical methods, i.e., coefficient determination (*R*^2^) and standard error (S.E.) for both calibration and validation datasets, were used to select the best-obtained model [38]. The *R*^2^ is the proportion of the variance in the dependent variable that is predictable from the independent variables and can be found with Equation (4). Meanwhile, the standard error of the regression represents the average distance that the observed values fall from the regression line, which can be calculated using Equation (5).

(4) $$R^{2}=\frac{\sum_{i}{\left({\hat{y}}_{i}-\bar{y}\right)}^{2}}{\sum_{i}{\left(y_{i}-\bar{y}\right)}^{2}}$$

(5) $$SE=\sqrt[{2}]{\frac{\sum_{i}{\left({\hat{y}}_{i}-y_{i}\right)}^{2}}{n-1}}$$

where ${\hat{y}}_{i}$ represents predicted value, $y_{i}$ represents the reference value and $\bar{y}$ is the mean of the reference values. The number of samples is symbolized with *n*. All data analysis processes were conducted using MATLAB software (The Math Works, Natick, MA, USA, R2019a). The workflow for model development was summarized and presented as Figure 2 (developing model).

### 2.5. Model Testing

To evaluate the applicability of the obtained model, the spectral data of 1365 soybean seeds belonging to 65 soybean varieties not initially involved in the model construction were collected using both instruments (FT-IR and FT-NIR). Then, the prediction values of the targeted components were calculated by multiplying the obtained regression coefficient with the seed spectral data in the new data set. On the other hand, after acquiring the spectral data, the soybean seeds were ground and sent for chemical analysis to examine the content of the targeted components. The coefficient determination (*R*^2^) and the standard error (SE) were then calculated to evaluate the model performance by using Equations (4) and (5), respectively. The summaries of the steps for model testing was presented in Figure 2 (testing model).

## 3. Results and Discussion

### 3.1. Spectral Data Interpretation

**Figure 3:**
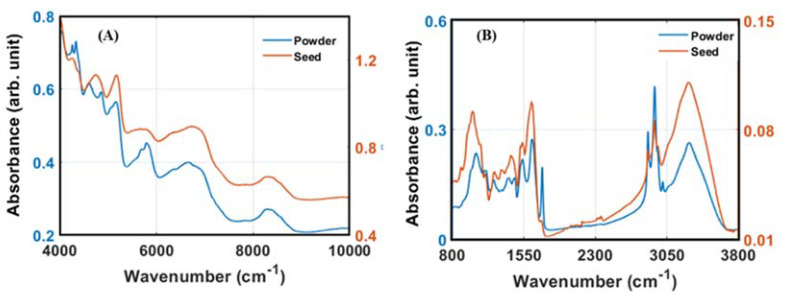
The typical pattern of raw spectra of soybean acquired using FT-NIR (**A**) and FT-IR (**B**).

The characteristics of the FT-NIR and FT-IR spectra for the soybean (Figure 3) represent some functional groups associated with the main components of the soybean, such as protein, carbohydrates, lipid, and moisture. The NIR spectra arise due to the vibration energy change between the fundamental (ground) state to the higher-order level or the combination bands. For the soybean sample, the near spectra can be highlighted as follows. The bands range from 5200 to 5100 cm^−1^, corresponding to the O–H vibration’s first overtone or combination mode, while the band between 5600 and 5000 cm^−1^ can be assigned to the vibration of C–H functional groups (first overtone and HC=CH form) related to the fatty acid component. Protein, the main soybean component, can be associated with the band between 5000 and 4500 cm^−1^, where the N–H and C=O stretching were detected [26]. The absorption bands ranging from 5720 to 6030 are related to the C–H first overtone region that correlated with the carbohydrates [39].

The FT-IR spectra (Figure 3B), consistent with the vibration in the primary energy level and the first excited vibration, can be basically separated into two waveband ranges. The fingerprint region (1200–900 cm^−1^) is the first range where the stretching of C–O, C–C, and C–O–C can possibly be identified. The stretching vibration of N–H that characterize protein arises around 1650 cm^−1^, while the asymmetric and symmetric CH2 and CH3 closely related to fatty acid were found at the band range of 3040 to 2850 cm^−1^ [40]. Figure 2 also revealed a straight line in the band range between 2600 to 2100 cm^−1^, indicating that no information can be obtained from this band range. Thus, this range of band was excluded for model development.

### 3.2. Reference Values Analysis

The result of the chemical data analysis for isoflavones and oligosaccharides was presented in Table 1.

Isoflavones are the essential flavonoid in soybean and have many health benefits, especially their ability to delay the menopausal period for women. In total, twelve types of isoflavones that belong to the three main types of isoflavones (daidzein, genistein, and glycitein) can be evaluated in the examined samples. However, the aglycone form (daidzein, genistein, glycitein) and two acetyl glucoside forms (acetyl genistin, acetyl glycitin) were only available at a low concentration level (below 50 µg/g) and had a narrow range of concentration. Therefore, the prediction model in this study was only developed for seven types of isoflavones and total isoflavones content, which was calculated by summing the content of individual isoflavone types detected in every soybean sample. The statistical data of the isoflavones, including the number of samples used for model development, are presented in Table 1. Overall, the concentration range obtained in this study coincided with the result presented by Berhow et al. [41]

Oligosaccharides were the next soybean chemical content observed in this study. Sucrose was the major oligosaccharide evaluated in the examined soybean samples and comprised about 50–55% of the total oligosaccharides in every sample. The galacto-oligosaccharides from the raffinose family (stachyose and raffinose) were observed around 36 to 40%, while other types of oligosaccharides (verbascose, maltose glucose, and fructose) only can be found in trace amounts in some examined soybean samples. Overall, this result was similar to the result reported by Hollung et al. [42] when evaluating carbohydrate content in Brazilian soybean. Therefore, due to the limited data of some individual oligosaccharide types, in this study, the prediction model was only developed for total oligosaccharides and three major types of observed oligosaccharides in the examined soybean samples.

### 3.3. Prediction Model of Soybean Chemical Components

#### 3.3.1. Isoflavones Model

The chemical composition of each bean within a single sample may differ due to different maturity stages and seed sizes. Thus, the spectral data of 21 seeds in a single sample were averaged, resulting in one spectrum representing one soybean sample. On the other hand, the result from the chemical analysis revealed that the soybean biochemical composition differs among the variety, which affects the number of spectral data used to develop a model for individual isoflavones types. The detail of the data can be seen in Table 1.

Our result, presented in Table 2, shows that it is possible to predict total isoflavones, as well as individual isoflavones in soybeans. We obtained the determination value for the calibration model (*R*^2^*_c_*) over 0.8 and an error of about 0.32 mg/g or equal to 14% of the total isoflavones content in the examined sample. Even though the *R*^2^*_c_* values for individual isoflavones were acceptable (over 0.7), the error was relatively high (over 15%), affecting the model confidence. The obtained models developed based on seed spectral data of FT-IR had a lower performance than the FT-NIR model, which is indicated by lower determination coefficients values (*R*^2^*_c_*, *R*^2^*_p_*) and higher error values (*SEC*, *SEP*). Meanwhile, the powder model of FT-IR showed comparable performance to FT-NIR. The FT-IR sample holder only allows measuring samples on a tiny area, which allows the possibility of generating inconsistent spectral data, particularly for inhomogeneous samples. Though a total of seven data preprocessing methods were used in this study, only the preprocessing methods that attained the highest performance are presented in Table 2 for brevity.

Previous studies related to the application of NIR and MIR to predict isoflavones in soybean were confined by the sample number and the concentration range of isoflavones in the examined sample. Therefore, multiple linear regression analyses have been applied to NIR spectral data to predict the content of isoflavones in whole and powder soybeans using only 48 samples [43]. In addition, by using the cross-validation method, the previous study obtained an acceptable model, denoted by *R*^2^ over 0.74 and 0.94, for individual isoflavones and total isoflavones for powder samples, respectively. The results also revealed an excellent model based on the spectra of whole soybean seeds (group of soybean) to predict total isoflavones (*R*^2^: 0.90). However, it failed to develop models for individual forms of isoflavones based on the spectra of the whole bean. Wang et al. [44] developed a model to predict isoflavone content in kudzu using NIRs based on 88 samples data and presented a consistent model. Berhow et al. [41] reported an excellent calibration model to predict total isoflavones with an *R*^2^ over 0.90 using more than 700 spectral data of ground soybean samples. For all mentioned reports, no report presented an acceptable model to predict the individual form of isoflavones in an intact seed using NIR or MIR. Therefore, our study obtained an acceptable model for predicting total isoflavones as well as individual types of isoflavones using a large amount of data using NIR and MIR spectra of a single seed.

**Figure 4:**
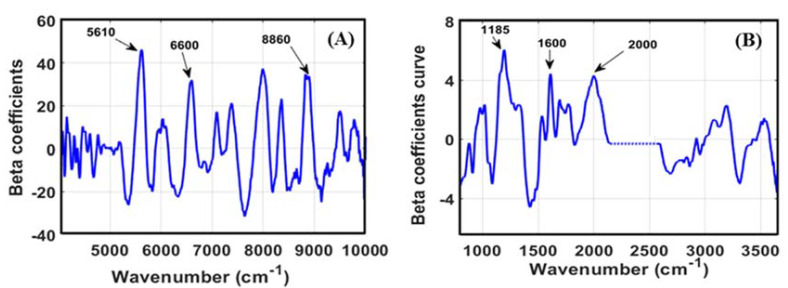
The regression coefficient of the PLSR model for isoflavones prediction using FT-NIR (**A**) and FT-IR (**B**).

In terms of PLS-R, one of the essential stages of applying NIRs and MIRs for developing a prediction model is identifying the specific waveband, which contributes significantly to the model performance. In this study, several bands in the NIR region can be identified as influential bands for isoflavones prediction (Figure 4A), which was also reported by previous research. The bands around 5600 cm^−1^ (1780 nm) and 6600 cm^−1^ (1515 nm) were reported by Zhang et al. [27] to have significant contributions to flavonoid and isoflavonoid prediction. These signals correspond to the stretching vibration’s first overtone of C–H. Another band that is considered influential is around 8860 cm^−1^ (1120 nm), which can be associated with the second overtone vibration of C-H from the aromatic ring structure [45].

Figure 4B locates a specific band of FT-IR spectral data for isoflavone detection. All of the marked bands corresponded to the unique structure of isoflavones, which contain two aromatic rings. The bands around 1185 cm^−1^ (8430 nm) and 2000 cm^−1^ (5000 nm) were assigned to C–H’s bending vibration and the combination band of aromatic structure. The last significant band was around 1600 cm^−1^ (6250 nm), corresponding to the unique aromatic ring bonding C=C–C [46].

#### 3.3.2. Oligosaccharides Model

The oligosaccharides prediction models based on FT-NIR and FT-IR spectral data were presented in Table 3. The result revealed that the FT-NIR was a promising method to predict total oligosaccharides and three main types of short-chain carbohydrates in soybeans using two different sample types: seeds and powder. Furthermore, the statistical parameters obtained based on the FT-NIR spectroscopy technique showed a good agreement between the prediction values and the reference values of the powder sample, indicated by the *R*^2^*_c_* values over 0.75 and low error (below 1%) for all of the evaluated chemicals. The seed-based model also exhibited an acceptable result, proven by low standard error values for both *SEC* and *SEP* (lower than 1%), representing a minimum difference between prediction and reference values. In addition, the results of the FT-NIR method were slightly better than those of FT-IR, denoted by higher *R*^2^*_p_* values and lower standard error values (*SEP*) for both sample types.

Carbohydrate is a macro component and one of the essential nutrients for humans. Thus, many previous research studies evaluated the robustness of NIR and MIR to predict this component in agriculture and food products. The effectiveness of NIR to predict carbohydrates in foxtail millet has been reported by Chen et al. [47] The full cross-validation method using 82 samples was applied to create a model and resulted in an excellent performance (*R*^2^) over 0.9 and low RMSE (below 0.8%). Ferreira et al. [39] used NIR spectral data of 82 varieties of Brazilian soybeans to predict the concentration of dietary fiber and obtained a high-performance model (*R*^2^) of 0.80 with an RMSEP of 0.86%. In addition, the sucrose content in soybean has also been successfully predicted by Choung [48]. However, most research was constrained by a limited number of samples, and the spectral data were acquired based on the powder sample, which is more homogenous. This current study developed models to predict total oligosaccharides as well as three main short-chain carbohydrates in soybean based on the seed spectral data. In addition, more reference values were also used to produce a more precise prediction model. Even though the obtained model revealed a lower performance, it could demonstrate the possibility of predicting oligosaccharides in soybean using an intact seed sample.

**Figure 5:**
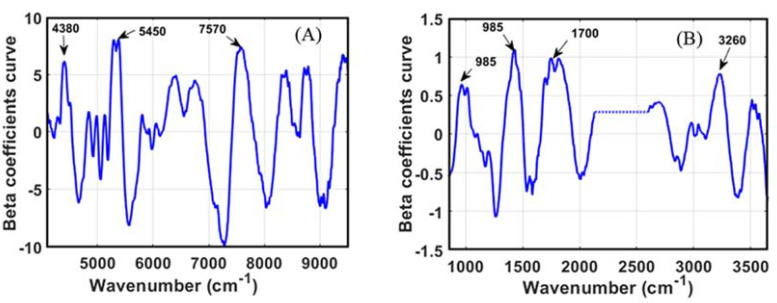
The beta coefficients curve for oligosaccharides prediction in soybean: (**A**) FT-NIR, (**B**) FT-IR.

The highly correlated bands of evaluated components can be identified based on the regression coefficients. Figure 5A presents the NIR beta coefficients to predict soybean oligosaccharides, revealing three important peaks. The peak around 4380 cm^−1^ (2280 nm) refer to the C–H stretch and CH_2_ deformation combination belonging to starch, while the peak around 5450 cm^−1^ (1820 nm) was assigned to the stretching of O–H or C–H bond’s second overtone that possibly comes from the cellulose structure. The last pointed band, around 7570 cm^−1^ (1300 nm), was closely related to the vibration of the C–H second overtone/combination from the CH_2_ structure [45]. Masithoh et al. [24] reported that bands at 4264 and 4380 cm^−1^ were influential bands for glucose. Other research reported by Fereira et al. [39] pointed out that the band at 5400 contributed to the model development based on NIR spectra for soybean dietary fiber. Oligosaccharides are part of carbohydrates; thus, their fundamental chemical structure is similar to monosaccharides and polysaccharides. Hence, the influential bands identified in this study coincide with the previous studies that evaluated carbohydrates components.

The regression coefficients to develop an oligosaccharides model using FT-IR spectroscopy are shown in Figure 5B. The most significant bands to determine oligosaccharides can be identified at 985, 1430, 1700, and 3280 cm^−1^. The bands between 900 and 1100 can be associated with the stretching and bending vibration of C–C and C–O bonds. The bands around 1700 cm^−1^ represent the crystal water in raffinose pentahydrate, while the bands that arise at 3260 can be assigned to the O–H stretching vibration [49].

### 3.4. Testing Model

Testing the model was a crucial part of ensuring the model’s performance and evaluating the obtained model’s applicability. This study developed a prediction model for isoflavones and oligosaccharides for total content as well as individual types value. Since the general concept of spectroscopy relies on the chemical bond vibration of the molecular structure of a compound, thus the biological compounds with similar chemical structures result in identical vibrational frequencies under the IR spectral range [50]. Hence, the influential bands obtained from the model analysis could not clearly be confirmed to originate from a specific individual type of component. In the same manner, the prediction result can be derived from specific kinds or mixed types (total) of examined components. Previous research by Schoonjans et al. [51] reported that compounds with similar chemical structures have been classified in the close class by using the hierarchical upgma-clustering method based on the IR spectral data.

In our study, individual types of examined components have the same chemical structure backbone. Hence, the influential bands obtained from the model analysis could not clearly be confirmed to originate from a specific type of component. From this point of view, testing the model for total components is more reasonable than individual types ones. Furthermore, the evaluation of isoflavones and oligosaccharides for the commercial application was only carried out for the total component instead of individual types. In addition, this research also emphasized predicting soybean components using an intact seed sample. Hence, the testing procedure was only performed to the seed-based model of the total content of evaluated components.

On the other hand, the results obtained from the chemical analysis showed that four soybean varieties contained isoflavones lower than 0.50 mg/g, and one contained over 5.0 mg/g. Therefore, to maintain the concentration range within the range of the model, we excluded those data. The contents of the targeted components and the number of samples used for the testing procedure are presented in Table 4.

**Figure 6:**
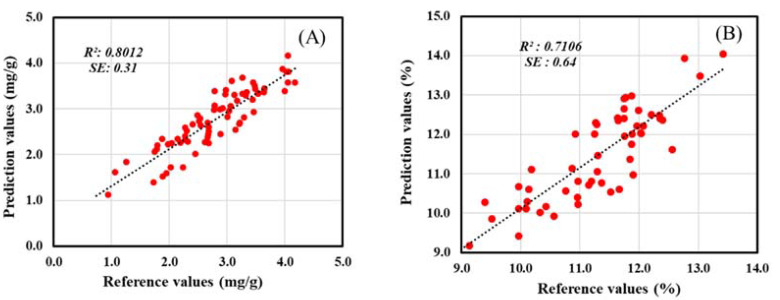
The distribution of the prediction data correlated with reference for the testing procedure using FT-NIR technique: (**A**) total isoflavones; (**B**) total oligosaccharides.

Statistical analysis of testing results showed a promising FT-NIR-technique-based model, presenting a similar *R*^2^ and error to the calibration model (Table 5). Meanwhile, the FT-IR technique presented *R*^2^ lower than the calibration model. The distribution of the prediction values and their correlation with the reference values for the FT-NIR technique can be seen in Figure 6.

Overall, the FT-NIR demonstrated better results than FT-IR on predicting isoflavones and oligosaccharides in soybean seeds. NIR radiation can penetrate the sample deeper than IR, which is beneficial for evaluating an agricultural product that is mostly categorized as an inhomogeneous sample. According to William and Noris [52], a model with *R_p_*^2^** values between 0.66 to 0.82 can be categorized as a model that can be used for sample screening.

## 4. Conclusions

Using an intact seed sample, FT-NIR and FT-IR spectroscopic techniques were investigated to determine the concentration of isoflavones and oligosaccharides in soybeans. In addition, the possibility of predicting the different types of targeted components was also investigated to explore the potential of these techniques to measure microcomponents of agricultural products. In total, 6510 seeds were selected randomly from 310 soybean samples, and their spectral data were collected using both instruments to develop a calibration and validation model. The testing model was carried out using 1365 seeds that belong to 65 soybean varieties, which were not involved in the model construction. The result showed that FT-NIR spectroscopy combined with the PLSR was a promising method predicting total isoflavones and oligosaccharides using intact soybeans seed, presenting a performance prediction model (*R*^2^*_p_*) of 0.80 and 0.72, respectively. The results of the testing model also demonstrated good performances, which were close to the calibration model. Meanwhile, FT-IR spectroscopy also shows an acceptable result, even though the performance (*R*^2^*_p_*: 0.73 and 0.70) was lower than the FT-NIR technique. The results of this fundamental study can be used as basic knowledge to develop a seed-sorting machine based on chemical components.

## Figures and Tables

**Table 1:** The statistical data of targeted soybean components used for reference value of the prediction model.

Components	Number of Samples	Mean ± SD	Max	Min
***Isoflavones (mg/g)***				
Daidzin	289	0.129 ± 0.057	0.317	0.018
Genistin	289	0.136 ± 0.043	0.250	0.039
Glycitin	265	0.042 ± 0.018	0.112	0.012
6-O-Malonyl daidzin	310	0.665 ± 0.271	1.403	0.110
6-O-Malonyl genistin	310	1.080 ± 0.375	2.028	0.200
6-O-Malonyl glycitin	280	0.147 ± 0.061	0.376	0.005
Acetyl daidzin	270	0.065 ± 0.021	0.123	0.020
Total isoflavones	310	2.320 ± 0.77	4.339	0.728
***Oligosaccharides (%)***				
Sucrose	310	6.001 ± 1.210	8.257	2.889
Stachyose	310	2.885 ± 0.517	4.067	1.781
Raffinose	310	1.124 ± 0.133	1.456	0.806
Total oligosaccharides	310	11.131 ± 1.645	14.643	6.879

**Table 2:** The PLSR statistical model for predicting total isoflavones and individual forms of isoflavones in soybeans using FT-NIR and FT-IR techniques.

Components (Preprocessing Method)	FT-NIR					FT-IR				
	*R* ^2^ *c*	*SEC*	*R* ^2^ *p*	*SEP*	*LVs*	*R* ^2^ *c*	*SEC*	*R* ^2^ *p*	*SEP*	*LVs*
***Seed***										
Daidzin (MN/MN)	0.74	0.03	0.72	0.03	25	0.72	0.03	0.70	0.03	25
Genistin (RD/RD)	0.73	0.02	0.70	0.03	25	0.70	0.02	0.67	0.03	25
Glycitin (MN/MN)	0.78	0.01	0.76	0.01	25	0.73	0.01	0.72	0.01	25
6-*O*-Malonyl daidzin (MN/MN)	0.77	0.14	0.75	0.12	25	0.72	0.15	0.70	0.20	25
6-*O*-Malonyl genistin (MN/MN)	0.79	0.17	0.77	0.18	25	0.71	0.19	0.70	0.29	25
6-*O*-Malonyl glycitin (MN/MN)	0.75	0.03	0.71	0.03	25	0.70	0.03	0.70	0.03	25
Acetyl daidzin (SNV/SNV)	0.76	0.01	0.73	0.01	23	0.71	0.02	0.68	0.02	22
Total isoflavones (MN/MN)	0.80	0.32	0.80	0.30	25	0.74	0.29	0.73	0.30	25
***Powder***										
Daidzin (RD/MN)	0.77	0.03	0.75	0.03	22	0.79	0.02	0.78	0.02	22
Genistin (RD/MN)	0.81	0.02	0.76	0.03	23	0.84	0.02	0.73	0.02	22
Glycitin (MN/MN)	0.76	0.01	0.74	0.01	24	0.74	0.01	0.70	0.01	22
6-*O*-Malonyl daidzin (MN/MN)	0.83	0.11	0.73	0.13	22	0.83	0.11	0.74	0.15	22
6-*O*-Malonyl genistin (MN/MN)	0.83	0.16	0.77	0.17	22	0.88	0.14	0.77	0.19	23
6-*O*-Malonyl glycitin (MN/MN)	0.73	0.03	0.72	0.03	24	0.78	0.03	0.74	0.03	24
Acetyl daidzin (SNV/SNV)	0.77	0.01	0.75	0.01	24	0.77	0.01	0.76	0.01	24
Total isoflavones (MN/MN)	0.92	0.21	0.84	0.33	25	0.92	0.21	0.84	0.33	25

*R*^2^*c*: coefficient determination of calibration; *SEC*: standard error of calibration; *R*^2^*p*: coefficient determination of prediction; *SEP*: standard error prediction; RD: raw data; MN; mean normalization; SNV: standard normal variate.

**Table 3:** The PLSR statistical model for predicting total oligosaccharides and short-chain carbohydrates in soybean using FT-NIR and FT-IR techniques.

Components (Preprocessing Method)	FT-NIR					FT-IR				
	*R*^2^*c*	*SEC*	*R* ^2^ *p*	*SEP*	*LVs*	*R* ^2^ *c*	*SEC*	*R* ^2^ *p*	*SEP*	*LVs*
***Seed***										
Sucrose (RD/MN)	0.72	0.71	0.70	0.75	19	0.72	0.67	0.71	0.68	19
Stachyose (RD/SNV)	0.70	0.28	0.66	0.29	19	0.67	0.30	0.66	0.33	19
Raffinose (SNV/SNV)	0.72	0.06	0.70	0.07	20	0.68	0.07	0.66	0.08	20
Total soluuble Carb (SNV/MN)	0.72	0.80	0.70	0.82	18	0.70	0.88	0.70	0.95	18
***Powder***										
Sucrose (RD/MN)	0.83	0.55	0.75	0.67	19	0.73	0.62	0.74	0.64	19
Stachyose (RD/SNV)	0.77	0.24	0.70	0.28	19	0.66	0.29	0.67	0.30	19
Raffinose (SNV/SNV)	0.77	0.06	0.72	0.06	20	0.73	0.07	0.72	0.07	20
Total soluuble Carb (SNV/MN)	0.78	0.74	0.75	0.80	18	0.73	0.87	0.72	0.84	18

*R*^2^*c*: coefficient determination of calibration; *SEC*: standard error of calibration; *R*^2^*p*: coefficient determination of prediction; *SEP*: standard error prediction; MN: mean normalization; RD: raw data; SNV: standard normal variate.

**Table 4:** Statistical data of the targeted components for testing model.

Components (Unit)	Number of Varieties	Mean ± SD	Max	Min
Total isoflvones (mg/g)	60	2.799 ± 0.736	4.176	0.946
Total oligosaccharides (%)	65	11.317 ± 0.938	13.419	9.135

SD: standard deviation.

**Table 5:** The statistical result of the testing model for predicting isoflavones and oligosaccharides in soybean seeds.

Components	N Seeds	N Varieties	*R*^2^**	*SE*
***FT-NIR***				
Total Isoflavones	1260	60	0.80	0.31
Total oligosaccharides	1365	65	0.71	0.64
***FT-IR***				
Total Isoflavones	1260	60	0.71	0.35
Total oligosaccharides	1365	65	0.68	0.81

## Data Availability

Not applicable.
